# Surgically treated metacarpal fractures in adults: a study with 3286 cases based on the Swedish National Quality Registry for Hand Surgery

**DOI:** 10.1186/s12891-026-10161-z

**Published:** 2026-07-08

**Authors:** Eva Lundqvist, Emelie Björkholm Wiktorell, Karolina Grännö, Marianne Arner, Lars B. Dahlin

**Affiliations:** 1 Faculty of Medicine and Health, Örebro University, Örebro, Sweden; 2 Department of Hand Surgery and Orthopedics, Örebro University Hospital, Örebro, Sweden; 3 Department of Clinical Science and Education, Karolinska Institutet, Stockholm, Sweden; 4 Department of Hand Surgery, Södersjukhuset Hospital, Stockholm, Sweden; 5 Department of Translational Medicine-Hand Surgery, Lund University, Malmö, Sweden; 6 Department of Hand Surgery, Skåne University Hospital, Malmö, Sweden

**Keywords:** Metacarpal fractures, Surgical techniques, Patient reported outcomes, Patient reported outcome measures, QuickDASH, National patient register, Trauma, Hand surgery, Fixation, Osteosynthesis

## Abstract

**Background:**

No international consensus regarding optimal non-surgical or surgical treatment of metacarpal fractures exists. Surgical outcome using patient-reported outcome measurements (PROMs) from national registers with large surgically treated population have rarely been reported. We present demographics and patient-reported outcomes in relation to fixation methods for surgically treated metacarpal fractures, using data from the Swedish National Quality Registry for Hand Surgery (HAKIR).

**Methods:**

In a retrospective analysis of a prospective collected dataset, patients aged ≥ 18 years with surgically treated metacarpal fractures registered in HAKIR during 2010–2022 were included (fracture types currently unknown), excluding reoperations and fractures associated with other injuries. PROMs (QuickDASH and HQ-8 routinely used) were collected at 3 and 12 months postoperatively. Data were compared with Chi-squared, Mann-Whitney, Kruskal-Wallis or Wilcoxon signed-rank tests and PROM changes over time were expressed as effect size.

**Results:**

In 3,286 analyzed cases, male patients were more frequently treated than females. Males were significantly younger than females (*p* < 0.001). Outcome, measured with QuickDASH, showed significant improvement from 3 (median: 15.9, interquartile range [IQR]: 6.8–31.8) to 12 months (median: 8.0, IQR: 2.3–20.5) (*p* < 0.001) with no significant differences between fractures in thumb, fingers, and multiple fractures at 12 months. QuickDASH scores at 12 months showed no significant differences between fixation methods. Outcome between open and closed fractures did not differ. K-wire/cerclage (61%) was most frequently used fixation method (plate fixation (16%), screw fixation (11%) with a significant variation between hospitals, e.g., variation K-wire/cerclage use: 29%-80% (*p* < 0.001). At 12 months, only HQ-8 item weakness showed significantly better outcome for screws without plating compared to K-wire/cerclage (*p* = 0.048).

**Conclusion:**

Based on national register data from a larger surgically treated population, good patient-reported outcome is expected one year after surgery for isolated metacarpal fractures with K-wire/cerclage being most common surgical fixation method. Despite notable national variation in fixation methods, likely influenced by currently unknown fracture types and possibly other confounding factors, no overall differences in outcome based on the PROM QuickDASH were observed. However, less weakness was noted 12 months postoperatively following screw fixation.

**Supplementary Information:**

The online version contains supplementary material available at 10.1186/s12891-026-10161-z.

## Background

Metacarpal fractures are common, both regarding hand fractures [1, 2] and fractures in general [1, 3], and with described differences in demographic patterns [4–7]. This is crucial from a societal perspective given the risk of sick leave and consumption of pain medication [2, 8]. Despite one of the used systems for classifications of the metacarpal fractures Arbeitsgemeinschaft für Osteosynthesefragen/Orthopedic Trauma Association (AO/OTA) classification [9], and a further division in different types [10], there is no international consensus for the optimal treatment of metacarpal fractures [11], even if a favorable prognosis often is achieved [4]. Stable, isolated, and simple metacarpal fractures are usually conservatively treated [12], because a non-operative approach facilitates early mobilization [13]. Concerning metacarpal fractures that require surgery, percutaneous pinning with Kirschner wires (K-wires) [14] may be preferred for simplicity, compared to lag-screw fixation, plate fixation, and external or intramedullary fixation.

Surgical management of metacarpal fractures vary between surgeons with no universally adopted guidelines for selection of surgical technique [4, 11, 15, 16]. A surgical intervention, which gives and maintains adequate stability, while still allows for early active rehabilitation, is probably chosen if surgery is needed. Recently, data indicate that, despite a metacarpal shortening, unrestricted mobilization in patients with a single displaced spiral and/or oblique metacarpal shaft fracture have a similar outcome as those patients surgically treated [17]. However, patient-specific factors, such as age, overall health status and individual functional demands, are also important aspects in decision-making [3, 18, 19]. Nevertheless, knowledge about surgery for and outcome in metacarpal fractures, based on patient-reported outcome measures (PROMs) from large national registers, are limited [20]. Different PROMs, often those that in a large population evaluate hand and arm function in general (like QuickDASH) or more detailed symptoms and abilities (like Hand Questionnaire-8; HQ-8; hakir.se), can be utilized in such studies with their own advantages and disadvantages. In addition, register-data provides a substantial number of different fracture types which are treated according to the surgeon’s decision in real life clinical practice; thereby the heterogeneity is in part considered. We hypothesize that variations in fracture and patient characteristics, and surgical treatment options are present, that affect outcomes measured by the PROMs QuickDASH and HQ-8 at 12 months post-surgery for a cohort of surgically treated metacarpal fractures in the Swedish National Quality Registry for Hand Surgery (HAKIR), and that an association exists between surgical technique and PROMs [21].

## Methods

**Fig. 1:**
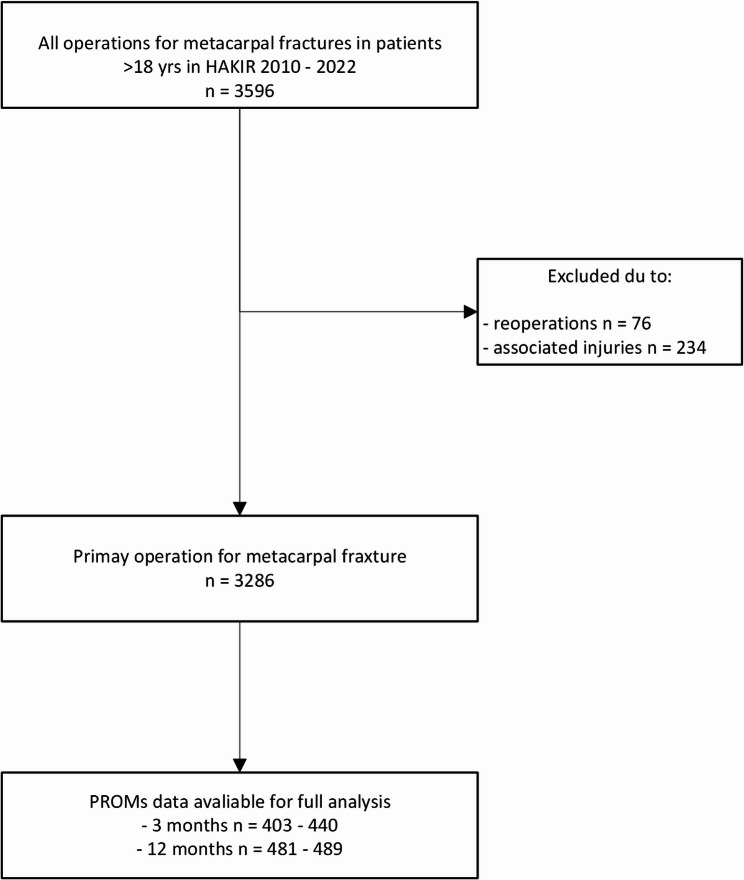
STROBE flow chart describing the inclusion and exclusion process

In a retrospective analysis of a prospective collected dataset, all primary surgically treated isolated and multiple metacarpal fractures using different osteosyntheses, registered in HAKIR [22] from the start of the registry on February 1 2010 to December 31 2022 were included. Cases were identified via the ICD-10 diagnostic codes [23] for isolated first metacarpal, isolated other metacarpal, and multiple metacarpal fractures (S622, S623, and S624, respectively) in combination with the NOMESCO surgical codes [24] for K-wire/cerclage fixation (NDJ49), screw fixation (NDJ79), external fixation (NDJ29) and combined fixation methods (NDJ89). Patients aged 18 years or older were included in this study [21]. Reoperations (e.g. due to complications or removal of osteosynthesis material) and metacarpal fractures with associated injuries were excluded (Fig. 1).

All surgeries performed at the seven specialized hand surgery departments in Sweden are registered in HAKIR, with a present coverage of over 95% [22]. PROMs are issued via web surveys to all patients before surgery and at 3 and 12 months postoperatively. Response rates are continuously monitored, and have varied between 25% and 50% [22].

The HAKIR questionnaire consists of two parts: the HQ-8 questionnaire and the short version of the Disabilities of the Arm, Shoulder and Hand questionnaire (QuickDASH) [25]. QuickDASH consists of 11 questions graded 1–5 on a Likert scale, and responses are used to calculate a total score of 0–100, with a higher score representing more severe disability. QuickDASH is not specific to the operated hand or to hand dominance [26, 27].

The HQ-8 questionnaire comprises 8 validated Likert-scale questions assessing various symptoms: pain on load, pain on motion without load, pain at rest, stiffness, weakness, numbness, cold sensitivity, and ability to perform daily activities [25, 28]. Each symptom is graded from 0 to 100, with a higher score representing more severe problems. These scores are not merged into a total score; instead, the HQ-8 complements QuickDASH with more specific information about the operated hand. Three questions from the HQ-8 questionnaire relevant for fractures were selected for further analysis; namely pain on load, stiffness, and weakness, which reflect different aspects of residual problems following a metacarpal fracture.

Data included in the analysis were: age, sex, hospital, fixation method, PROMs, which metacarpal bone was affected (thumb, finger, or multiple fractures), and whether the fracture was open or closed (fractures with missing data were considered to be closed).

### Ethical approval and consent to participate

According to Swedish law, all patients must be adequately informed before registration and given the opportunity to opt out if they wish, but active individual consent is not required for registration. The opt-out process requires contact with local or national registry coordinator for total exclusion from the registry, and such process for this is described in the patient information. This study was approved by the Regional Ethical Review Board in Stockholm, Sweden, and the National Ethical Review Board (refs: 2017/2023-31, 2019 − 00880 and 2021 − 00902). The study was performed according to the Helsinki Declaration [29, 30].

### Statistics

Demographic data are presented as number and percentage of cases, or as median and interquartile range (IQR). The Shapiro-Wilk test was used to assess normal distribution (data not shown). Due to a non-normal distribution in almost all parameters and the use of ordinal data, the data are presented as median and IQR. A chi-squared test was used for comparison of categorical data. The Mann-Whitney test or the Kruskal-Wallis test was used for comparison of ordinal data between independent groups, and the Wilcoxon signed-rank test was used for dependent comparisons. For these non-parametric tests, Hodges–Lehmann estimates of the median difference together with 95% confidence intervals were calculated. A t-test was used for group comparison of continuous data. Missing data were handled with pairwise exclusion. P-values less than 0.05 were considered to be statistically significant. When multiple comparisons were performed, Bonferroni post-hoc corrections were used, and adjusted p-values are presented. Data were analyzed using version 29.0.0 of IBM SPSS Statistics, and visualizations were produced with version 2024.12.0 of RStudio for macOS.

## Results

During the study period, 3,596 operations for metacarpal fractures were registered in HAKIR. Cases with reoperations (*n* = 76) or associated injuries (*n* = 234) were excluded, leaving 3,286 metacarpal fractures for analysis (Fig. 1). Of these 3,286 metacarpal fractures, 692 (21.1%) affected the first metacarpal, 2,219 (67.4%) were single metacarpal index, long, ring, and little finger fractures, and 375 (11.4%) were multiple metacarpal fractures. There were 101 (3.1%) cases of open fracture.

**Fig. 2:**
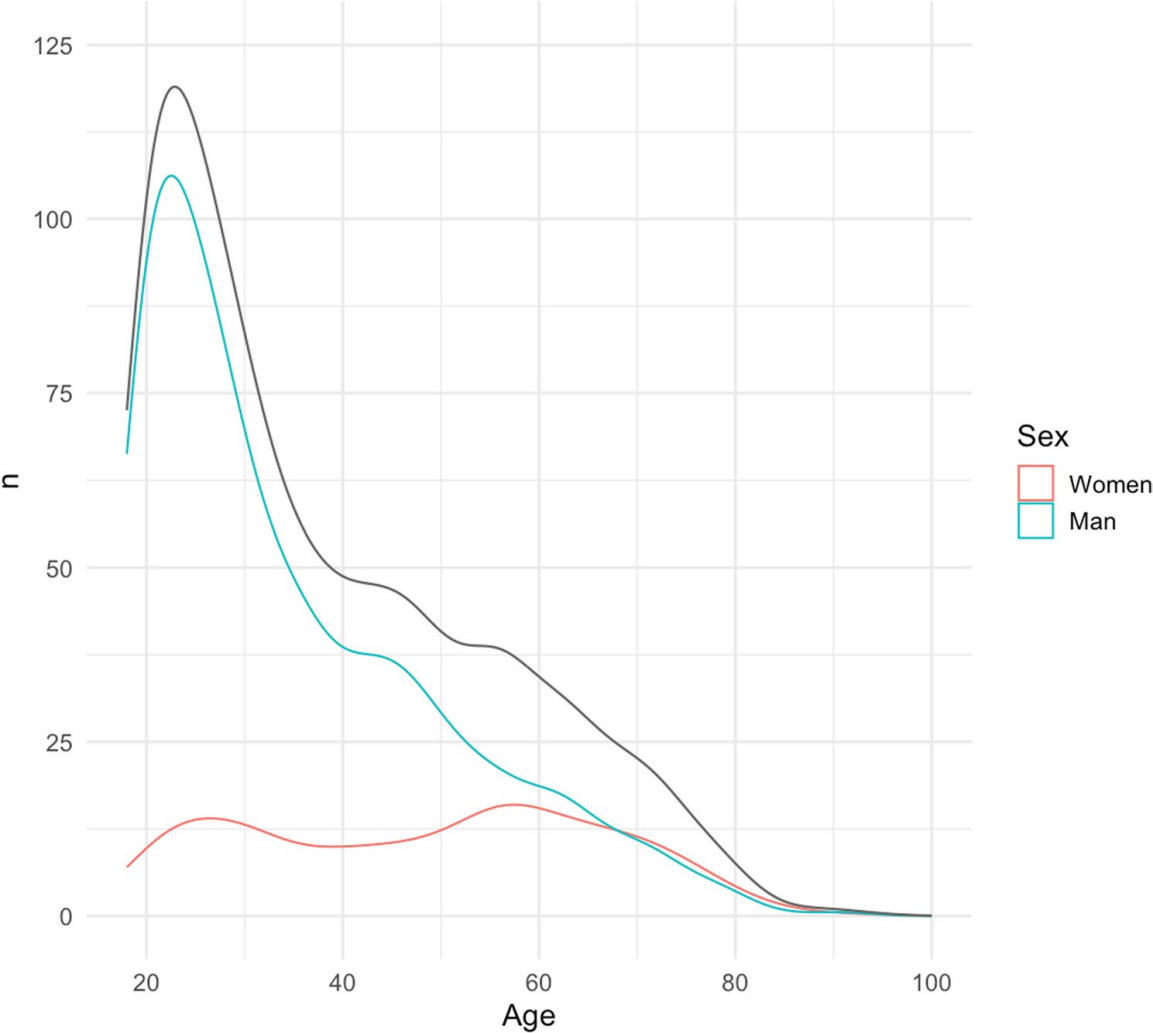
Total incidence curve by age of patients with closed and open isolated and multiple metacarpal fractures affecting the thumb and fingers reported to the Swedish National Quality Registry for Hand Surgery [22]. Red = women, blue = men, black = total. y-axis = fractures/year of age, x-axis = age in years

Sex and age characteristics are presented in Table 1. Of the 3,286 fractures, 2,098 affected the right hand (63.8%), 1,180 (35.9%) involved the left hand, and 8 (0.2%) were bilateral or an unknown side. In the total group of patients, 2,812 (85.6%) were aged 18–59 and 474 (14.4%) were aged 60 or older. There were significantly more male patients than female patients, and the male patients were younger than the female patients (*p* < 0.001) (Table 1, Fig. 2).

**Table 1:** Sex and age characteristics of the patients

	*n* (%)	Age, median (IQR)
Female	763 (22.3)	50 (31–63)
Male	2523 (76.8)	30 (23–44)
Total	3286 (***p*** < 0.001)	33 (24–50) (***p*** < 0.001)

Patients with closed and open isolated and multiple metacarpal fractures affecting the thumb and fingers reported to the Swedish National Quality Registry for Hand Surgery [22]

*IQR *interquartile range

###  Patient-reported outcomes

PROMs data were available for 403–440 cases at 3 months and 481–489 cases at 12 months (Table 2). The QuickDASH score significantly improved from 15.9 (6.8–31.8) at 3 months to 8.0 (2.3–20.5) at 12 months (Table 2), which is probably not a minimal clinical important difference (MCID; see Discussion). Moreover, all the HQ-8 items except the question on cold sensitivity showed a statistically significant improvement between 3 and 12 months (Table 3); levels that may not be MCID (MCID not available for HQ-8; see Discussion).

**Table 2:** Comparison of responders vs. non-responders of QuickDASH at 3 and 12 months

	3 months			12 months		
	Responders (*n* = 438)	Non-responders (*n* = 2848)	*p*-value	Responders (*n* = 484)	Non-responders (*n* = 2802)	*p*-value
Age	42 (27–57) yrs	32 (24–49) yrs	< 0.001	40 (26–57) yrs	32 (24–49) yrs	< 0.001
Female	143 (32.6%)	620 (21.8%)	< 0.001	155 (32.0%)	608 (21,7%)	< 0.001
Open fracture	17 (3,9%)	84 (2.9%)	NS	15 (3.1%)	86 (3.1%)	NS
Type of surgery	239 (54.6%) K-wire or cerclage	1752 (61.5%) K-wire or cerclage	< 0.001	267 (55.2%) K-wire or cerclage	1724 (61.5%) K-wire or cerclage	0.001
Metacarpal I/II-V/multiple	107/290/41	585/1929/334	NS	112/320/52	580/1899/232	NS

Characteristics of responders vs non-responders of the PROM questionnaire QuickDASH in HAKIR. Data are given as median (interquartile range). Age is compared using the Mann-Whitney test, the other variables are compared using Chi2-test

*NS *non-significant

**Table 3:** Patient-reported outcome measures (PROMs) at 3 and 12 months

PROMs	3 months (*n* = 403–440)	12 months (*n* = 481–489)	Cohen’s d	*P*-value ^a^
Pain on load (HQ-8 1)	28 (10–50)	16 (0–30)	0.44 (0.31–0.57)	< 0.001
Pain on motion without load (HQ-8 2)	10 (0–20)	0 (0–10)	0.30 (0.17–0.43)	< 0.001
Pain at rest (HQ-8 3)	0 (0–10)	0 (0–10)	0.14 (0.01–0.27)	0.013
Stiffness (HQ-8 4)	20 (10–50)	10 (0–30)	0.38 (0.25–0.51)	< 0.001
Weakness (HQ-8 5)	30 (10–50)	10 (0–30)	0.48 (0.35–0.62)	< 0.001
Numbness (HQ-8 6)	1 (0–20)	0 (0–10)	0.25 (0.12–0.37)	< 0.001
Cold sensitivity (HQ-8 7)	2 (0–30)	0 (0–22)	0.06 (-0.08–0.19)	NS (0.28)
Ability to perform daily activities (HQ-8 8)	10 (0–30)	0 (0–20)	0.39 (0.26–0.52)	< 0.001
QuickDASH	15.9 (6.8–31.8)	8.0 (2.3–20.5)	0.51 (0.38–0.65)	< 0.001

Patients with closed and open isolated and multiple metacarpal fractures affecting the thumb and fingers reported to the Swedish National Quality Register for Hand Surgery [22]. Data are given as median (interquartile range)

*NS *non-significant, *CI *confidence interval

^a^ Wilcoxon signed-rank test

The only significant sex differences were that at 12 months, female patients had a higher QuickDASH score than male patients (11.4 vs. 6.8; *p* = 0.031), while male patients had a higher score for the HQ-8 question on numbness than female patients (but the same median of 0; *p* = 0.045) (Additional file 1; for MCID see Discussion).

The QuickDASH score at 12 months was higher for women aged ≥ 60 (14.8, IQR: 4.6–36.9) than for both younger women (9.1, IQR: 2.3–20.5) (*p* = 0.028) with a Hodges–Lehmann median difference of 4.55 points (95% CI 0.00–9.09), and men aged ≥ 60 (5.7, IQR: 0.0–27.3) (*p* = 0.042) with a median difference of 4.55 points (95% CI 0.00–10.23; for MCID see Discussion). There were no statistically significant differences when comparing men aged under 60 years (6.8, IQR 0.0–18.2) either to women in the same age group, or to men aged ≥ 60 [31].

Patients with different fracture locations (thumb, fingers, or multiple metacarpals) showed no statistically significant differences in either QuickDASH or the selected relevant HQ-8 questions (pain on load, stiffness, and weakness) at either 3 months or 12 months. Furthermore, no statistically significant differences in these outcomes were seen between open and closed fractures (results not shown).

### Osteosynthesis methods

The most frequently used surgical treatment was K-wire/cerclage (60.6%), followed by plating (16.4%) and fixation with screws (11.3%) (Table 3). In the total group, K-wire/cerclage was more commonly used in male patients (*p* < 0.001). When the data were dichotomized by age (18–59 vs. ≥60 years), there was no sex difference in the older population. In the younger group of patients, K-wire/cerclage was significantly less commonly used for women than for men, but it was still the most frequently used fixation method (*p* < 0.001) (Table 4).

**Table 4:** Surgical treatment by sex and age group

Age group (years)	Sex	K-wire or cerclage	Plating	Screws without plating	Other fixation methods	Total
18–59	Female	267 (50.7%)	90 (17.1%)	106 (20.1%)	64 (12.1%)	527
	Male	1,402 (61.4%)	408 (17.9%)	216 (9.5%)	259 (11.3%)	2,285
	Total	1,669 (59.4%)	498 (17.7%)	322 (11,5%)	323 (11.5%)	2,812
≥ 60	Female	161 (68.2%)	22 (9.3%)	27 (11.4%)	26 (11.0%)	236
	Male	161 (67.6%)	29 (12.2%)	23 (9.7%)	25 (10.5%)	238
	Total	322 (67.9%)	51 (10.8%)	50 (10.5%)	51 (10.8%)	474
All	Female	428 (56.1%)	112 (14.7%)	133 (14.7%)	90 (10.8%)	763
	Male	1,563 (62.0%)	437 (17.3%)	239 (9.5%)	284 (11.3%)	2,523
	Total	**1,991 (60.6%)**	549 (16.7%)	372 (11.3%)	374 (11.4%)	3,286

Patients with closed and open isolated and multiple metacarpal fractures affecting the thumb and fingers reported to the Swedish National Quality Register for Hand Surgery [22]. Data are given as *n* (%). Chi2 test , 18–59 years: *p* <0.001; ≥60 years: non-significant (*p* = 0.584); all *p* <0.001

There was a statistically significant difference in the choice of fixation methods between isolated metacarpal fractures in the thumb or fingers or in multiple metacarpals (*p* < 0.001), with K-wire/cerclage being more common in thumb fractures, and “other fixation methods” being more common in multiple fractures.

There was no statistically significant difference in choice of fixation method between open and closed fractures (results not shown). However, a statistically significantly higher proportion of open fractures was found in cases with multiple metacarpal fractures (5.6% vs. 2.7%, *p* = 0.011).

There were statistically significant between-hospital differences in treatment methods, with the rate of K-wire/cerclage varying from 29.2% to 79.5% (*p* < 0.001, Table 5).

**Table 5:** Surgical treatment by hospital

	K-wire or cerclage	Plating	Screws, without plating	Other fixation methods	Total
Hospital A	598 (55.4%)	197 (18.2%)	188 (17.4%)	97 (9.0%)	1080
Hospital B	359 (61.3%)	98 (16.7%)	78 (13.3%)	51 (8.7%)	586
Hospital C	308 (63.1%)	56 (11.5%)	39 (8.0%)	85 (17.4%)	488
Hospital D	372 (79.5%)	28 (6.0%)	7 (1.5%)	61 (13.0%)	468
Hospital E	80 (29.2%)	111 (40.5%)	37 (13.5%)	46 (16.8%)	274
Hospital F	131 (73.2%)	9 (5.0%)	19 (10.6%)	20 (11.2%)	179
Hospital G	123 (70.7%)	40 (23.0%)	2 (1.1%)	9 (5.2%)	174
Other sites	20 (54.1%)	10 (27.0%)	2 (5.4%)	5 (13.5%)	37
Total	1991 (60.6%)	549 (16.7%)	372 (11.3%)	374 (11.4%)	3286

Patients with closed and open isolated and multiple metacarpal fractures affecting the thumb and fingers reported to the Swedish National Quality Register for Hand Surgery [22]. Chi2 test: *p* <0.001

### Outcome based on QuickDASH and HQ-8 questionnaires

QuickDASH scores at 3 months after surgery were significantly higher for K-wire/cerclage (20.5; IQR: 9.1–34.1) compared to plating (11.4; IQR: 2.3–20.5) (*p* = 0.003; for MCID see Discussion) and screws without plating (11.4; IQR: 4.6–25.0) (*p* = 0.013), indicating inferior outcome. QuickDASH at 3 months did not significantly differ between “other fixation methods” and the other treatments (15.4; IQR: 6.8–31.8). At 12 months, there were no statistically significant between-method differences in QuickDASH.

All three selected HQ-8 questions (pain on load, stiffness, and weakness) showed a statistically significant difference between techniques at 3 months, but at 12 months the only remaining significant difference was for weakness, which was significantly better for screws without plating compared to K-wire/cerclage (*p* = 0.048) (Additional file 2; for MCID see Discussion).

## Discussion

In this retrospective analysis of a prospective collected dataset, we report on surgically treated metacarpal fractures in more than 3000 adults (≥ 18 years) with data collected from a Swedish national quality registry, where two PROMs (i.e., QuickDASH and HQ-8) are routinely used, and complement each other, to evaluate outcome of surgical procedures in fractures as well as in various hand disorders [32–34]. In general, the data showed that the outcome, based on the QuickDASH scores, at 12 months is not associated with the used surgical method, involved thumb or fingers, the presence of multiple fractures, or whether the fracture was open. Within the country, various fixation methods, likely influenced by currently unknown fracture types and other confounding factors as occupation, comorbidities, handedness and mechanism(s) of injury, were employed with K-wire/cerclage as fixation method being most prevalent. The involvement of the dominant (right) hand [1, 35] may have a potential impact on both the professional life and leisure activities, which is reported in another registry study using other outcome measures at one year [20]. PROM data indicated that at 3 months post-surgery, patients rated their upper limb function (QuickDASH score) slightly worse and some aspects of hand symptoms (HQ-8) better if treated with plating and screws compared to patients treated with K-wire/cerclage, but at 12 months post-surgery only weakness remained significant in favor for fixation with screws. It should be noted that the presently used fixation methods may likely be influenced by the currently unknown fracture types and some other confounding factors. As expected, a higher frequency of surgically treated metacarpal fractures in younger men are observed in accordance with a recent Swedish registry study [20], but the sex difference diminishes with increasing age.

In national registers, PROMs have to be selected that are suitable and simple, but still valid and appropriate, for all types of injuries and diseases. In HAKIR, QuickDASH and HQ-8 were initially selected and these two PROMs complement each other [32]. In view of the observed statistically observed differences, one need to consider the clinical relevance of the found differences for QuickDASH and HQ-8. This is validated by detecting and referring to the minimal clinically important difference. According to Franchignoni et al. [36], MCID for the total QuickDASH score is considered to range from 16 to 20. The MCID for HQ-8 has not been assessed, but we considered a present difference of at least 10 points to be clinically relevant. At one year, the median score for the HQ-8 item “pain on load” was 16, indicating that some patients had residual pain problems on load. Nevertheless, our patients demonstrated a significant and progressive improvement in PROMs from 3 to 12 months postoperatively, regardless of whether the injury was open or closed, whether there were a single fracture or multiple fractures, or which finger (or thumb) was affected. However, the improvement from 3 to 12 months does not reach MCID for any of the PROMs. The clinical interpretation is therefore unclear, but one may interpret the findings that the improvement of symptoms and functions are limited during the first year. Even if the statistically found improvements were significant and did not reach the definition of a MCID, it still underscores the importance of clearly informing patients about the necessity of rehabilitation throughout the entire first postoperative year [37].

Fracture types or complexity of the fractures may be two factors in the choice of fixation method, but unfortunately the factors are not registered in the national register HAKIR. Neither are data on comorbidities, smoking, surgeon experience, nor mechanism(s) of injury registered, which all potentially, together with the type or complexity of the fractures, may affect both the choice of surgical method and outcome. The surgical method was chosen “in real clinical life” based on the surgeons´ experience, the fracture type, the possibility to achieve stability, the need for the surgical trauma in relation to the soft tissue, the time for an appropriate time and probably also grounded on local traditions. Despite introduction of screws and plates and screws over the years [11], K-wire/cerclage is still the most frequently used fixation method. More men than women in the younger age group underwent surgery using this fixation method. Fixation with K-wire/cerclage induces minimal tissue damage, but may still ensure a sufficient stable fixation of isolated metacarpal fractures [38, 39], and probably also less postoperative pain [40].

The differences in fixation method between hospitals are likely due to local traditions, variations in surgical indications depending on fracture types, and the experience of the hand surgeons involved. These circumstances are probably more important than the case mix. The main advantage of plate and screws (or only screws) is the rigid fixation [14], which has been highlighted in a few prospective studies of complex metacarpal fractures resulting in excellent functional outcomes [15]. Plating, based on a larger surgical exposure with pronounced tissue exposure and risk of creating soft tissue adhesions, may lead to increased postoperative stiffness compared to e.g., K-wire/cerclage [14, 39]. However, the present patient-reported stiffness did not differ between fixation using plating compared to pinning. Overall, in addition to the aforementioned surgical considerations, it is essential also to account for health economic factors to reduce surgical costs, to decrease outpatient follow-up expenses, and to maximize the societal economic benefits associated with return to work [41]. The register data does not include information about the extent of rehabilitation resources, which also are crucial aspects of treatment pf metacarpal fractures [37]. With this in mind, different types of comorbidities may also impact the outcome of hand injuries and metacarpal fractures in particular through the ability of the patient to handle the injury and subsequent treatment, i.e., their coping strategies [42]. However, these perspectives were not possible to investigate in the present study but may be evaluated through linking various national registers, including the Swedish fracture register which includes fracture severity [43]. Nevertheless, a lesson that can be learned from a clinical perspective, is that the absence of the present long-term differences in PROMs suggests that surgical decisions should prioritize fracture stability, soft-tissue preservation, and rehabilitation rather than the fixation method alone [37].

Based on the observed national differences regarding the choice of surgical method, it is crucial to investigate how patients perceive the outcomes following different surgical procedures. It should be noted that the present patients all were surgically treated at specialized university hospitals (and for a minority of patients at one private hand unit) in contrast to patients included in other national registers [20]. Some difficulties were found with weakness, stiffness, and in performing daily activities at 3 months among patients treated with K-wire/cerclage compared to those treated with plate and screws, but the differences generally disappeared at 12 months (only a minor difference in weakness). These data indicate the fracture and tissues heal over time and that it is essential to encourage patients to continue exercising during follow-up to regain full hand function. Older women rated their mobility worse after 12 months compared to their male peers and younger women, which may be attributable to a selective sample, meaning that only the most severe fractures in older women are operated on, and that the group as a whole may be undertreated. The increased risk in the older female age group is probably due to biological changes, including increased bone fragility, osteoporosis, and balance problems [1, 8]. In Sweden, the current life expectancy for women is 84.7 years [44], which is a result of a good standard of living, higher education, and advances in healthcare [45]. This increases the opportunities for high physical activity well into old age and thus possibly also pave the way for an increased risk of fractures. However, elderly women may also experience limitations in hand function after other traumatic injuries [31].

Like in other hand fractures, a sex difference, with an overrepresentation of younger men, was found [1, 7, 8, 20, 46], which may be a result of their work situation characterized by high physical strain compared to women [8] or related to a high degree of physical capacity and risk-taking behavior among young men [8]. HAKIR lacks information on occupation, cause of injury or any other socioeconomic data, but we assume that demographic factors and socioeconomic status in Sweden is associated with an increased risk of fractures like in other hand injuries [47].

There are several limitations of the present study, such as that we only focused on surgically treated metacarpal fractures and that only patients operated by hand surgeons were included in contrast to another registry study [20]. The national HAKIR-register only includes data on surgical management of injuries and disorders and only university hand surgical departments and some private hand surgery units were connected to the register most of the investigated study period. Furthermore, we did not have information about the individual types of metacarpal fracture (AO/OTA- classification), comorbidities, the extent of tissue injuries (see exclusion criteria) as well as the experience of the surgeons; information that may lack in national registers, but data from such registers represent “the clinical real life”. Another weak point that is often seen in registers, is that we could not elucidate the role of missing data and study dropout (see data on responders versus non-responders; a common pattern in register studies). Thus, the major limitation is the high level of missing PROMs data, i.e., response rate, that limits the interpretation of causality in the data as well as the suitability to perform multiple imputations and/or multivariable regression analyses. However, evaluation of causality is a complex issue and require use of the Bradford Hill criteria [48]. Future projects in our register research will aim to address the issue of response rate. Future research on metacarpal fractures should investigate how fracture type influences the indication for surgical intervention and the choice of new fixation methods [49–51] and possibly also prospectively monitor postoperative outcomes over several years, although the outcome in the present study at one year showed generally good outcome. Prospective randomized studies are another alternative [17], but should also involve health economic evaluations; a crucial aspect in the health care sector with limited resources.

Other limitation in this study is the absence of preoperative PROMs and the psychometric properties of the PROMs in the registry. However, these PROMs have been selected in the registry since they have been validated in both elective and emergency surgery [32]. Preoperative PROMs in acute trauma, such as metacarpal fractures, presents several methodological challenges. Preoperative PROMs are subject to substantial recall bias and several other methodological challenges. In emergency settings, patients typically experience pain, distress, and acute loss of function, while PROMs are designed to capture a patient’s stable perception of symptoms and function. In acute trauma, these perceptions are transient and dominated by the immediate effects of the injury rather than the patient’s pre-injury state and is therefore not available in the registry.

## Conclusions

Surgically treated isolated metacarpal fractures, being common in younger men, is expected to have a good patient-reported outcome, based on PROMs with the inherited limitations of registry studies, one year after surgery for these metacarpal fractures. Despite a notable national variation in fixation methods, likely influenced by currently unknown fracture types and possibly other confounding factors, where fixation with K-wire/cerclage is most frequently used, no between-method association were observed in QuickDASH score at 12 months postoperatively. 

## Supplementary Information

Supplementary Material 1.

## Data Availability

The datasets used and analyzed during the current study are available from the corresponding author on reasonable request.

## Abbreviations

AO/OTA: Arbeitsgemeinschaft für Osteosynthesefragen/Orthopedic Trauma Association
HAKIR: Swedish National Quality Registry for Hand Surgery
K-wire: Kirschner wire
MCID: Minimal Clinically Important Difference
PROM: Patient-reported outcome measure
QuickDASH: Disabilities of the Arm, Shoulder and Hand questionnaire, short version
