# Supplementary material for: Surgically treated metacarpal fractures in adults: a study with 3286 cases based on the Swedish National Quality Registry for Hand Surgery
Source: BMC Musculoskelet Disord. 2026 Jul 8;27:607. doi: 10.1186/s12891-026-10161-z (PMC13371171; doi:10.1186/s12891-026-10161-z)
Supplement: Supplementary file 1 — Supplementary Material 1. [file 12891_2026_10161_MOESM1_ESM.docx]

# Additional files

Additional file 1: Patient-reported outcome measures (PROMs) at 3 and 12 months, divided by sex, of metacarpal fractures.

|  | Women  n=143–156 | Men  n=294–333 | p-value^a^ |
| --- | --- | --- | --- |
| **PROMs at 3 months** |  |  |  |
| Pain on load (HQ-8 1) | 30 (10–50) | 27 (10–50) | NS (0.42) |
| Pain on motion without load (HQ-8 2) | 10 (0–20) | 10 (0–20) | NS (0.85) |
| Pain at rest (HQ-8 3) | 0 (0–10) | 0 (0–10) | NS (0.26) |
| Stiffness (HQ-8 4) | 30 (10–50) | 20 (10–43) | NS (0.20) |
| Weakness (HQ-8 5) | 30 (10–50) | 30 (10–50) | NS (0.89) |
| Numbness (HQ-8 6) | 0 (0–10) | 1 (0–20) | NS (0.63) |
| Cold sensitivity (HQ-8 7) | 1 (0–40) | 4 (0–30) | NS (0.86) |
| Ability to perform daily activities (HQ-8 8) | 15 (0–30) | 10 (0–30) | NS (0.63) |
| QuickDASH | 15.9 (6.8–34.1) | 13.6 (6.8–29.6) | NS (0.19) |
|  |  |  |  |
| **PROMs at 12 months** |  |  |  |
| Pain on load (HQ-8 1) | 10 (0–30) | 19 (0–30) | NS (0.12) |
| Pain on motion without load (HQ-8 2) | 0 (0–10) | 0 (0–10) | NS (0.95) |
| Pain at rest (HQ-8 3) | 0 (0–10) | 0 (0–10) | NS (0.16) |
| Stiffness (HQ-8 4) | 10 (0–40) | 10 (0–30) | NS (0.22) |
| Weakness (HQ-8 5) | 20 (0–30) | 10 (0–30) | NS (0.36) |
| Numbness (HQ-8 6) | 0 (0–10) | 0 (0–10) | **0.045**^b^ |
| Cold sensitivity (HQ-8 7) | 0 (0–20) | 1 (0–30) | NS (0.08) |
| Ability to perform daily activities (HQ-8 8) | 0 (0–20) | 0 (0–20) | NS (0.42) |
| QuickDASH | 11.4 (2.3–22.7) | 6.8 (0.0–18.1) | **0.031** |

Data are presented as median (interquartile range). NS=non-significant.
^a^Mann-Whitney test.
^b^HQ-8 question 6: inferior results for women.

Additional file 2: Patient-reported outcome measures (PROMs) for different surgical treatments of metacarpal fractures.

|  | PROMs at 3 months | | | | | | | | PROMs at 12 months | | | | | | | |
| --- | --- | --- | --- | --- | --- | --- | --- | --- | --- | --- | --- | --- | --- | --- | --- | --- |
|  | QuickDASH n=438 | adj. p‑value | HQ-8 Q1 n=438 | adj. p‑value | HQ-8 Q4 n=440 | adj. p‑value | HQ-8 Q5 n=537 | adj. p‑value | QuickDASH n=484 | adj. p‑value | HQ-8 Q1 n=488 | adj. p‑value | HQ-8 Q4 n=488 | adj. p‑value | HQ-8 Q5 n=489 | adj. p‑value |
| K-wire or cerclage | 20.5  (9.1–34.1) |  | 30 (10–50) |  | 25 (10–50) |  | 30 (10–60) |  | 6.8 (0.0–25.0) |  | 19 (0–30) |  | 10 (1–36) |  | 17 (0–30) |  |
| Plating | 11.4  (2.3–20.5) | **0.003** | 20 (10–33) | **0.010** | 20 (0–40) | **0.037** | 20 (10–40) | **0.015** | 6.8 (1.1–13.4) | NS | 10 (0–30) | NS | 10 (0–30) | NS | 10 (0–30) | NS |
| Screws without plating | 11.4 (4.6–25.0) | **0.013** | 20 (0–30) | **<0.001** | 25 (10–50) | NS | 20 (10–40) | **0.019** | 6.8 (0.0–18.2) | NS | 10 (0–30) | NS | 10 (0–30) | NS | 10 (0–30) | **0.048** |
| Other fixations | 15.4 (6.8–31.8) | NS | 20 (10–42) | NS | 20 (10–50) | NS | 30 (19–40) | NS | 6.8 (2.3–19.3) | NS | 20 (1–35) | NS | 20 (0–30) | NS | 20 (0–33) | NS |

p-values obtained with the Kruskal-Wallis test. Statistical significance compared to K-wire/cerclage after Bonferroni post-hoc corrections. No other comparisons were statistically significant. NS=non-significant.
